# Heterotypic and homotypic continuity in psychopathology: a narrative review

**DOI:** 10.3389/fpsyg.2023.1194249

**Published:** 2023-06-15

**Authors:** Anna Maria Speranza, Marianna Liotti, Ilaria Spoletini, Alexandro Fortunato

**Affiliations:** ^1^Department of Dynamic and Clinical Psychology, and Health Studies, Sapienza University of Rome, Rome, Italy; ^2^Department of Medical Sciences, IRCCS San Raffaele Pisana, Rome, Italy

**Keywords:** psychopathology, homotypic continuity, heterotypic continuity, adolescence, childhood

## Abstract

Psychopathology is a process: it unfolds over time and involves several different factors. To extend our knowledge of such process, it is vital to understand the trajectories that lead to developing and maintaining a specific disorder. The construct of continuity appears very useful to this aim. It refers to the consistency, similarity, and predictability of behaviors or internal states across different developmental phases. This paper aims to present a narrative review of the literature on homotypic and heterotypic continuity of psychopathology across the lifespan. A detailed search of the published literature was conducted using the PsycINFO Record and Medline (PubMed) databases. Articles were included in the review based on the following criteria: (1) publication dates ranging from January 1970 to October 2022; and (2) articles being written in the English language. To ensure a thorough investigation, multiple combinations of keywords such as “continuity,” “psychopathology,” “infancy,” “childhood,” “adolescence,” “adulthood,” “homotypic,” and “heterotypic” were used. Articles were excluded if exclusively focused on epidemiologic data and if not specifically addressing the topic of psychopathology continuity. The literature yielded a total of 36 longitudinal studies and an additional 190 articles, spanning the research published between 1970 and 2022. Studies on continuity focus on the etiology of different forms of mental disorders and may represent a fundamental resource from both a theoretical and clinical perspective. Enhancing our understanding of the different trajectories beneath psychopathology may allow clinicians to implement more effective strategies, focusing both on prevention and intervention. Since literature highlights the importance of early detection of clinical signs of psychopathology, future research should focus more on infancy and pre-scholar age.

## 1. Introduction

Most adults with a psychiatric diagnosis have a history of psychopathological disorders during childhood and/or adolescence (Kim-Cohen et al., 2003). The presence of psychopathological symptoms in childhood increases the odds of having a mental disorder in adolescence or early adulthood up to threefold (Copeland et al., 2013). Since psychopathology is a complex process that unfolds over time (Cicchetti, 1984; Sroufe and Rutter, 1984), describing its developmental course is vital from both a diagnostic and prognostic point of view. However, different risk or protective factors may modify psychopathological trajectories during different life stages. Developmental age is characterized by multiple transformations involving several neurobiological changes, and numerous new acquisitions and tasks. These transformations shape both the emergent personality patterns and the overall symptomatic manifestations of individuals. Therefore, psychological functioning during childhood or adolescence requires a careful and complex assessment and a thorough understanding of its underlying dynamics. Such assessment must always consider how each diagnosis evolves over time, the modifications in symptoms expression linked to neurobiological changes, and the clinical manifestations associated with each specific life phase.

The parent–child relationship is an additional aspect that requires careful evaluation as it can function as either a protective or risk factor for development. Particularly, attachment – which forms the foundation for the ongoing patterns of development – plays a crucial role in this regard. Although not directly associated with psychopathology, it can trigger developmental processes that, when examined, contribute to a more thorough understanding of the continuity of disorders.

### 1.1. Definitions of continuity

The term “continuity” refers to the psychological structures, traits, or learned behaviors that may be detected from one developmental period to the next (Cicchetti, 1984; Sroufe and Rutter, 1984). It also refers to consistency, similarity, and predictability among behaviors or states over time (Siegel, 1999), both in typical and atypical development, and is defined by the presence of a common causal link (Costello et al., 2003) in both analogous and different clinical manifestations across the lifespan.

The term homotypic continuity is used when a psychiatric disorder is predictive of the same condition during subsequent assessment. In this case, similar traits or behaviors can be observed in different developmental phases and measured by the same set of indicators (*ibidem*). Conversely, the term heterotypic continuity refers to when a disorder predicts a different one later in time (Shevlin et al., 2017). In this case, behaviors or traits assume another phenomenological form over time (Kagan, 1969).

Studies on continuity may expand our knowledge of how psychopathology unfolds across the lifecycle and enhance our understanding of the etiology of mental disorders (Rutter and Sroufe, 2000). In addition, recognizing the developmental trajectories of different forms of psychopathology may be pivotal for implementing effective prevention strategies. Considering these issues, we reviewed the literature on homotypic and heterotypic continuity of psychopathology across the lifespan. To our knowledge, there is only another recent review investigating this topic (Zarrella et al., 2017), which has focused both on retrospective and prospective studies. In the present work, since retrospective studies usually emphasize the role of risk factors rather than previous diagnoses, we chose to focus mainly on longitudinal and prospective studies.

## 2. Methods

We conducted a detailed search of the published literature with a review of the PsycINFO Record and Medline (PubMed) databases. Articles were included in the review based on the following criteria: (1) publication between January 1970 and October 2022; (2) being written English language. For our purposes, we used various combinations of the following keywords: “continuity,” “psychopathology,” “infancy,” “childhood,” “adolescence,” “adulthood,” “homotypic,” “heterotypic.” Articles were excluded if exclusively focused on epidemiologic data and if not specifically addressing the topic of psychopathology continuity. This exclusion criterion was chosen because a review on prevalence rates of psychiatric disorders across developmental periods has been already published (Costello et al., 2011).

A first search was performed to identify articles specifically focused on homotypic and heterotypic continuity. A second search was conducted to extract records containing a source of information (i.e., data, models, or hypotheses) on topics that are conceptually related to continuity of psychopathology (in particular, attachment). Finally, we considered cross-references and other review articles reported in the papers previously collected.

All articles cited in this manuscript were judged by the Authors to be relevant and to meet the scientific and conceptual criteria listed.

The literature search resulted in a pool of 36 longitudinal studies and other 190 articles covering the published research between 1970 and 2022.

## 3. Homotypic and heterotypic continuity of internalizing disorders

Continuity of internalizing symptoms from early childhood to later ages has been extensively demonstrated (Briggs-Gowan et al., 2006; Snyder et al., 2017; Monk et al., 2021). Children with internalizing symptoms appear behaviorally inhibited, withdrawn, and prone to anxiety, depression, and somatic complaints. Internalizing disorders have been defined as based on “overcontrolled” symptoms (Kovacs and Devlin, 1998), indicating that they are inner-directed and linked to the attempt to preserve an excessive or maladaptive control of one’s states. A significant heterogeneity of symptoms across studies has been found (Tandon et al., 2009). Continuity patterns of internalizing disorders are summarized in Table 1.

**Table 1:** Continuity patterns of internalizing disorders.

Mental disorder	Developmental age	Study	Continuity pattern
Anxiety Disorders (AD)	preschool-age ➝ adolescence	Costello et al., 2003	AD ➝ AD AD ➝ DeD AD ➝CD
	childhood ➝ adolescence	Burke et al., 2005	AD ➝ DeD
		Huizink et al., 2006	AD ➝ SA
		Finsaas et al., 2018	AD ➝ AD AD ➝ DeD
		Copeland et al., 2013	AD ➝ AD AD ➝ DeD
	childhood ➝ adulthood	Copeland et al., 2009	AD ➝ DeD
		Huizink et al., 2006	AD ➝ SA
	adolescence ➝ adulthood	Pine et al., 1998	AD ➝ DeD
		Copeland et al., 2013	AD ➝ AD AD ➝ DeD
Generalized Anxiety Disorder (GAD)	childhood ➝ adolescence	Bittner et al., 2007	GAD ➝ CD OAD ➝ OAD OAD ➝ PA, DeD, CD
		Ferdinand et al., 2007	GAD ➝ GAD
Obsessive–Compulsive Disorder (OCD)	childhood ➝ adolescence	Ferdinand et al., 2007	OCD ➝ OCD
Anxiety Separation Disorder (ASD)	childhood ➝ adolescence	Bittner et al., 2007	ASD ➝ ASD
		Ferdinand et al., 2007	ASD ➝ ASD
Social Phobia (SoP)	childhood ➝ adolescence	Bittner et al., 2007	SoP ➝ SoP SoP ➝ OAD SoP ➝ ADHD
		Ferdinand et al., 2007	SoP ➝ SoP
	adolescence ➝ adulthood	Pine et al., 1998	SoP ➝ SoP
Simple Phobia (SiP)	adolescence ➝ adulthood	Pine et al., 1998	SiP ➝ SiP
Panic Disorder (PD)	childhood ➝ adolescence	Ferdinand et al., 2007	PD ➝ PD
Depressive disorders (DeD)	preschool-age ➝ adolescence	Fergusson and Woodward (2002)	DeD ➝ DeD DeD ➝ AD	
	childhood ➝ adolescence	Costello et al., 2003	DeD ➝ DeD DeD ➝ AD		
		Huizink et al., 2006	DeD ➝ SA
		Copeland et al., 2013	DeD ➝ DeD DeD ➝ AD DeD ➝ ADHD DeD ➝ SA DeD ➝ CD	
	childhood ➝ adulthood	Copeland et al., 2009	DeD ➝ AD
		Huizink et al., 2006	DeD ➝ SA
	adolescence ➝ adulthood	Pine et al., 1998	DeD ➝ AD
		Fergusson and Woodward (2002)	DeD ➝ DeD DeD ➝ AD DeD ➝ SA DeD ➝ suicidal attempts		Copeland et al., 2013	DeD ➝ DeD DeD ➝ AD
Major Depressive Disorder (MDD)	preschool-age ➝ school age	Luby et al., 2009	MDD ➝ MDD
	preschool-age ➝ adolescence	Gaffrey et al., 2018	MMD ➝ MDD
	adolescence ➝ adulthood	Rao et al., 1999	MDD ➝ DeD

AD, Anxious Disorders; ADHD, Attention-Deficit/Hyperactivity Disorder; ASD, Anxiety Separation Disorder; CD, Conduct Disorder; DeD, Depressive Disorder; GAD, Generalized Anxiety Disorder; MDD, Major Depressive Disorder; OAD, Overanxious Anxiety Disorder; OCD, Obsessive–Compulsive Disorder; ODD, Oppositional Defiant Disorder; PA, Panic Attacks; PD, Panic Disorder; SA, Substance Abuse; SiP, Simple Phobia; SoP, Social Phobia.

### 3.1. Anxiety disorders

A significant number of studies cover the area of anxiety disorders. Research has highlighted a significant homotypic continuity pattern for anxiety during developmental age (Costello et al., 2003; Finsaas et al., 2018). For example, separation anxiety disorder tends to remain stable during adolescence (Bittner et al., 2007) and adulthood (Osone and Takahashi, 2006). The same pattern seems to also characterize generalized anxiety disorder, social phobia, panic disorder, and obsessive–compulsive disorder (Ferdinand et al., 2007). More specifically, the presence of social phobia and other forms of simple phobia during adolescence is predictive of the same condition in adulthood (Fyer et al., 1995; Shaffer et al., 1996; Pine et al., 1998). Instead, childhood separation anxiety is predictive of a subsequent panic disorder (Pine et al., 1996), which tends to remain stable from adolescence to adulthood (Keyl and Eaton, 1990). It also predicts agoraphobia and generalized anxiety disorder in adulthood, with a U-shaped pattern showing a reduction of symptoms in middle childhood and a subsequent increase from early adolescence to young adulthood (Copeland et al., 2014). More generally, the presence of socially and emotionally anxious behaviors in childhood is a significant risk factor for anxiety disorders in adolescence and adulthood (Monk et al., 2021).

Homotypic and heterotypic continuity patterns of anxiety disorders were thoroughly investigated by the TRacking Adolescents’ Individual Lives Survey (TRAILS; Ferdinand et al., 2007) in early adolescents (10–12 years). Evidence for homotypic continuity was found for separation, social, and generalized anxiety in both genders, as well as for panic disorder in girls. This homotypic trajectory seems to be created by a combination of genetic, temperamental, and environmental factors, which leads to an anxious personality organization (Cohen et al., 2018). Such organization, in turn, seems to lead to different symptomatologic expressions during development in response to the challenges that characterize each phase (Rudolph et al., 2016).

Concerning heterotypic continuity, the presence of anxiety disorders during childhood is related to the development of depressive disorders during adolescence (Costello et al., 2003; Burke et al., 2005; Bittner et al., 2007) and adulthood (Pine et al., 1998; Copeland et al., 2009). These disorders have a strong heterotypic continuity, especially among girls, even when controlling for comorbidity between the two (Costello et al., 2003; Cummings et al., 2014). Research suggests that anxiety symptoms are an age-dependent manifestation of the same pathological pattern found in depression (Weissman et al., 2005). Genetic and twin studies support this hypothesis, suggesting a common genetic etiology with distinctive phenotypical expressions during different developmental phases and in response to different experiences (Silberg et al., 2001; Rice et al., 2004; Middeldorp et al., 2005; Waszczuk et al., 2014).

Anxiety separation disorder, generalized anxiety disorder (GAD), and panic disorder are the syndromes with a higher probability of transforming into a major depressive disorder (MDD) in adulthood (Wittchen et al., 2000; Rutter et al., 2006), with MDD and GAD having the highest level of shared genetic risk (Kalin, 2020). In addition, the presence of anxiety disorders during adolescence is associated with a higher risk of developing not only an anxiety disorder later in life but also depression, alcohol and substance abuse, suicidal behaviors, educational underachievement, and early parenthood (Woodward and Fergusson, 2001).

### 3.2. Depressive disorders

Similar to anxiety, depression also tends to remain stable over time. It is a strong predictor of the development of subsequent depression since preschool age (Luby et al., 2009, 2014). Depression in childhood or adolescence is a significant risk factor for both ensuing depressive disorders and a wide array of other forms of psychopathology (Avenevoli and Steinberg, 2001; Fergusson and Woodward, 2002; Costello et al., 2003; Kasen et al., 2009; Gaffrey et al., 2018); as such, it should be diagnosed as early as possible.

As already noted, depression and anxiety seem to have both high comorbidity rates (Cummings et al., 2014) and heterotypic continuity patterns (Cohen et al., 2018), suggesting the presence of a common etiology and a highly interconnected symptom structure (McElroy et al., 2018). Childhood depression shows a heterotypic continuity pattern with anxiety disorders during adolescence (Fergusson and Woodward, 2002; Costello et al., 2003; Copeland et al., 2009), while the presence of a major depressive episode in adolescence is strongly predictive of a subsequent anxiety disorder (Pine et al., 1998; Fergusson and Woodward, 2002; Kim-Cohen et al., 2003). Childhood depression is also predictive of a subsequent substance abuse disorder (Huizink et al., 2006) and higher suicidal risk in adulthood (Woodward and Fergusson, 2001; Fergusson and Woodward, 2002). Another longitudinal study has also shown heterotypic continuity patterns between childhood depression and pathological personality patterns, such as dependent, antisocial, passive-aggressive, and histrionic personality disorders (Kasen et al., 2001).

Depression shows a strong homotypic continuity from childhood to adolescence, which becomes less significant from teenage years to early adulthood (Copeland et al., 2013). Other studies (Cohen et al., 2018; Gaffrey et al., 2018) have revealed that children diagnosed with depression during pre-school years show a decrease in symptoms during school age, with a new exacerbation in adolescence. Morken et al. (2021) have shown that, during early adolescence, depressive symptoms become both more severe and less likely to reduce over time. Rao et al. (1999) have found that late adolescent women who had an MDD were significantly more at risk for depression later in life, as well as for difficulties in academic achievement and intimate romantic relationships. Children with a prepubertal onset have a higher risk of developing depression if an MDD diagnosis is present in the history of their mothers (Weissman et al., 1999). When depression is diagnosed during pre-school years, however, it remains highly predictive of post-pubertal depression even after controlling the effect of maternal depression (Gaffrey et al., 2018).

The homotypic pattern regarding depression is strongly influenced by other factors, such as socioeconomic status (Wickrama et al., 2012), exposure to stressors, and adverse life events (Rice et al., 2003). Lower exposure to biological changes and social stressors (Larson et al., 2002; Ge et al., 2006) positively impacts this trajectory, even if the contribution of both genetic and environmental factors seems to vary significantly during different life phases (Nivard et al., 2015).

## 4. Homotypic and heterotypic continuity of externalizing disorders

Externalizing disorders define a wide range of aggressive and disruptive outward-directed behaviors (Campbell et al., 2000), such as oppositional defiant disorders (ODD), attention-deficit/hyperactivity disorder (ADHD), conduct disorder (CD), and substance-related and addictive disorders (Greenspan and Wieder, 2006; American Psychiatric Association, 2013).

Continuity patterns of externalizing disorders are shown in Table 2.

**Table 2:** Continuity patterns of externalizing disorders.

Mental disorder	Developmental age	Study	Continuity pattern
Conduct Disorder (CD)	preschool-age ➝ adolescence	Lahey et al., 2002a	CD ➝ ODD CD ➝ ADHD CD ➝ DeD CD ➝ AD
	childhood ➝ adolescence	Gaffrey et al., 2018	CD ➝ CD
		Graham and Rutter (1973)	CD ➝ CD CD ➝ affective disorders
		Copeland et al. (2013)	CD ➝ SA
	childhood ➝ adulthood	Offord et al., 1992	CD ➝ CD CD ➝ affective disorders
		Costello et al., 2003	CD ➝ SA
Oppositional Defiant Disorder (ODD)	preschool age ➝ childhood	Wichstrøm et al., 2017	ODD ➝ CD
	childhood ➝ adolescence	Burke et al., 2005	ODD ➝ CD ODD ➝ AD ODD ➝ DeD
		Lahey et al., 2002a	ODD ➝ CD
		Copeland et al., 2013	ODD ➝ ODD
ADHD	preschool age ➝ childhood	Wichstrøm et al., 2017	ADHD ➝ AD
		Costello et al., 2003	ADHD ➝ ODD
		Burke et al., 2005	ADHD ➝ ODD
		Shevlin et al., 2017	ADHD ➝ internalizing disorders
		Copeland et al., 2013	ADHD ➝ ADHD ADHD ➝ ODD
		Obsuth et al., 2020	ADHD ➝ externalizing disorders
	preschool age ➝ adolescence	Finsaas et al., 2018	ADHD ➝ ADHD ADHD ➝ DBD
	childhood ➝ young adulthood	Vos et al., 2021	ADHD ➝ ADHD ADHD ➝ DeD

AD, Anxious Disorders; ADHD, Attention-Deficit/Hyperactivity Disorder; ASD, Anxiety Separation Disorder; DBD, disruptive behavior disorder; DeD, Depressive Disorder; GAD, Generalized Anxiety Disorder; MDD, Major Depressive Disorder; OCD, Obsessive Compulsive Disorder; PA, Panic Attacks; PD, Panic Disorder; SA, Substance Abuse.

Children with externalizing problems are at increased risk for juvenile delinquency, academic failure, and social maladjustment (King et al., 2004; Englund and Siebenbruner, 2012; Basten et al., 2016); more generally, externalizing problems in childhood predict future psychopathology (Egeland et al., 1996; Campbell et al., 2000; Hofstra et al., 2002). The presence of high levels of hyperactivity-impulsivity, non-compliance, physical aggression, and other externalizing behaviors can be detected from a very early age (Mesman et al., 2001; Lorber et al., 2015; Carbonneau et al., 2016; Biedzio and Wakschlag, 2018; Wiggins et al., 2018), and these tend to remain consistent during development, creating patterns of both heterotypic and homotypic continuity.

To better comprehend the continuity of externalizing disorders, Copeland et al. (2013) have conducted a meta-analysis using data from various longitudinal studies – namely, the Great Smoky Mountains Study (Costello et al., 1996), the Christchurch Health and Development Study (Fergusson et al., 1989) and the Dunedin Multidisciplinary Health and Development Study (Silva, 1990). They found that externalizing disorders tend to be more stable and to present higher homotypic transitions than internalizing disorders, confirming the results of several other studies (Graham and Rutter, 1973; Offord et al., 1992; Achenbach et al., 1995; Pine et al., 1998; Ferdinand et al., 2007). CD seems to possess a stable homotypic trajectory from pre-school to adolescence (Gaffrey et al., 2018). ADHD and ODD show a robust homotypic pattern from childhood to adolescence (Finsaas et al., 2018; Obsuth et al., 2020).

ODD is a predictor of CD during adolescence (Lahey et al., 2002a), and both ODD and CD during childhood predict ADHD during teenage years (Wichstrøm et al., 2017). A recent study showed that the course of ADHD is highly heterogeneous; its trajectory is mediated by the severity of symptoms in childhood, risk for depression, medication use, IQ levels, comorbidities, and functional impairment (Vos et al., 2021). Other authors have found that ADHD during childhood remains a significant predictor for later ODD (Costello et al., 2003) and antisocial disorder (Mannuzza et al., 2004). Furthermore, research has shown that the relationship between ODD and CD may be bidirectional, as ODD symptoms are significant predictors of CD symptoms, and the latter predict ODD symptoms (Lahey et al., 2002a; Burke et al., 2005). A sub-analysis from the Developmental Trends Study (Burke et al., 2005), in which 177 boys were followed-up yearly until age 18, revealed that ADHD is a significant predictor of ODD. Homotypic and heterotypic continuity patterns were observed with depression, overanxious disorder, ADHD, ODD, and CD (*ibidem*). The authors (Burke et al., 2005) argued that ODD symptoms are related to the presence of temperamental and/or interpersonal difficulties and emerging aspects of antisocial personality traits, while CD symptoms reflect behavioral control problems.

CD and ADHD are significant precursors of both substance abuse disorder in adolescence (Disney et al., 1999; Costello et al., 2003) and antisocial personality disorder (Biederman et al., 1996; Patterson et al., 2000; Rutter et al., 2006; Copeland et al., 2009). In addition, externalizing psychopathology is a predictor of early alcohol, nicotine, and cannabis consumption, as well as of regular experience with these substances (King et al., 2004). Reviewing the results of seven North American controlled prospective longitudinal studies, Cherkasova et al. (2021) found that children with ADHD show significant difficulties in school and occupational functioning and significantly lower mental and physical well-being levels. They also present higher rates of substance abuse, antisocial behavior, and other risk-taking behaviors, including suicide attempts. Overall, the majority (84%) of children with persistent ADHD also had a comorbid disorder by young adulthood, with more than half of them having two or more comorbidities (Cherkasova et al., 2021).

Continuity between externalizing disorders can be better understood by looking at the presence of dysregulated patterns (altered control of attention, emotion, impulse, and arousal). The role of the lack of emotional and behavioral regulation plays a significant role in aggression, defiance, delinquency, and substance abuse behaviors (Eisenberg et al., 2010). Some authors (Beauchaine and McNulty, 2013) have proposed that ADHD, ODD, CD, substance abuse, and antisocial personality disorder have a common matrix, resulting from complex longitudinal transactions between interdependent individual vulnerabilities (e.g., genetic, epigenetic, temperamental) and contextual risk factors (e.g., experiences within the caregiving system and the peer group). Other authors (Wakschlag et al., 2018) have proposed that externalizing behaviors can be considered a neurodevelopmental syndrome that remains stable across the lifespan. Twin and family studies have shown that externalizing disorders can be conceptualized as a single continuous dimension of vulnerability in which hereditary factors play a significant role (Schmitz and Mrazek, 2001; Krueger, 2002; Kendler et al., 2003; Hicks et al., 2004; Blonigen et al., 2005; Bornovalova et al., 2010). A longitudinal study has demonstrated that the developmental trajectory of externalizing disorder is influenced by a wide array of factors, regarding both hereditary and environmental influences (Petersen et al., 2015), which supports the hypothesis proposed by Beauchaine and McNulty’s (2013) hypothesis. However, further research is needed to clarify the interplay between externalizing disorders and whether a common causal link may explain the observed continuity patterns.

## 5. Cross-predictions between internalizing and externalizing disorders

There are also cross-predictions between internalizing and externalizing disorders (Costello et al., 2003). For example, the presence of a conduct disorder during childhood is predictive of anxiety and depression in adolescence (Graham and Rutter, 1973; Capaldi, 1992; Offord et al., 1992; Lahey et al., 2002a,b; Rutter et al., 2006). ODD during childhood is predictive of anxiety and depressive symptoms both in adolescence (Burke et al., 2005) and in adulthood (Kim-Cohen et al., 2003; Copeland et al., 2009), while ADHD during pre-school age is predictive of internalizing disorders during both school-age years (Wichstrøm et al., 2017) and adolescence (Shevlin et al., 2017). High levels of externalizing behaviors during childhood are predictive of internalizing symptoms later in life; changes in the symptomatology of one of the domains (externalization or internalization) often correspond to changes in the other (Gilliom and Shaw, 2004). Pesenti-Gritti et al. (2008) have found that high levels of externalizing symptoms predict subsequent co-existing internalizing problems in children.

Similarly, childhood anxiety and depression predict the development of externalizing symptoms. Anxious disorders during childhood are in heterotypic continuity with both conduct disorders (Costello et al., 2003; Bittner et al., 2007) and substance disorders (Huizink et al., 2006) during both adolescence and adulthood. A 21-year longitudinal study of a birth cohort of 1,265 children (Fergusson and Woodward, 2002) demonstrated that depression was a risk factor for developing nicotine dependence, alcohol abuse, or dependence in teenage years. Such heterotypic patterns may be explained considering the more significant impact of social and relational factors (e.g., interactions with peers) in adolescence, which could drive teenagers to express their distress through externalization. Externalizing behaviors, however, follow a different trajectory in boys and girls. Boys show more physically aggressive behaviors, whereas girls tend to show indirect aggression (Côté et al., 2007; Marmorstein, 2007). All forms of depressive disorders during developmental age seem to be associated with heightened levels of anxiety, substance abuse disorders, risky/criminal behaviors, and poor social functioning in adulthood (Copeland et al., 2018). These associations persisted when accounting for childhood comorbidities (such as anxiety and conduct disorder) and adverse experiences (*ibidem*). A 24-year longitudinal study from the Zuid-Holland population-based study (Reef et al., 2009) examined continuity patterns of internalizing and externalizing symptoms in children and adolescents (4–16 years): childhood aggression, delinquent behavior, and anxious and/or depressive symptoms are the strongest predictive factors for adult psychopathology (see also Hofstra et al., 2000; Althoff et al., 2010), while attention problems alone are not predictive of later difficulties (Reef et al., 2009). In addition, other studies have shown that anxiety and subsequent depression increase the risk for antisocial behavior (De Graaf et al., 2003; Burke et al., 2005; Diamantopoulou et al., 2010).

More recently, a study (Papachristou and Flouri, 2020) has shown that children with severe and persistent externalizing problems were more likely to develop a depressive disorder in early adolescence. After controlling for comorbidity with other conditions, Costello et al. (2003) found that continuity patterns between CD and depression remained significant for girls but not for boys. However, another study (Maughan et al., 2004) found an opposite trend. There is evidence that CD predicts depression, while depression is not predictive of antisocial behavior (Rutter et al., 2006). CD was also found to predict depression during early adolescence (Capaldi, 1992). The interconnections between behavioral and affective disorders could be understood by referring to the “failure model” (Patterson and Capaldi, 1990; Capaldi, 1992). According to this model, conduct problems lead to higher levels of interpersonal conflict, rejection, lack of support, and poor skills development, increasing the risk of subsequent depression and other forms of severe psychopathology. Studies have confirmed that difficulties in social interactions significantly predict depression (Patterson and Stoolmiller, 1991) and that peer rejection mediates the relationship between aggression and depression (Panak and Garber, 1992; Keiley et al., 2000; Kiesner, 2002).

Other authors have proposed that continuity patterns between externalizing and internalizing disorders can be explained through a shared etiopathogenetic factor. Recent studies (Finsaas et al., 2018; Waldman et al., 2021), for instance, have stressed the role played by the “irritability” factor, which can be considered a common characteristic among CD, ODD, childhood depression, and some forms of anxiety disorders during development. According to this model, the genetic or constitutional factors that influence children’s irritability levels in early childhood also predispose them to anxiety and depression in adolescence and/or adulthood. Frost et al. (2019), instead, have found that cortisol patterns moderate the course of internalizing and externalizing patterns from early childhood to adolescence: a steeper cortisol decline at age nine is predictive of higher internalizing symptoms at age twelve, while a blunted cortisol decline at the same age predicts higher externalizing problems Another etiological mechanism for externalizing disorders can also be found in environmental influences, such as maternal psychopathology (Jobs et al., 2019).

## 6. Continuity of other psychiatric disorders

In the last twenty years, dysregulation disorders (i.e., sleeping, eating, and sensory processing disorders) have received growing attention due to the importance of behavioral and sensory regulation for adjustment and well-being (Winsper et al., 2020). However, literature on the continuity of dysregulation disorders is still scarce. The few available data have provided interesting suggestions. Symptoms of dysregulations in various areas show a homotypic pattern, remaining stable from early childhood to pre-adolescence (Boomsma et al., 2006; Hemmi et al., 2011). For what concerns heterotypic continuity, a study (DeGangi et al., 2000) revealed that 95% of infants with moderate regulatory disorders develop subsequent motor, language, and cognitive disorders, as well as relational problems. In addition, there is growing evidence that sensory processing disorders in infancy predict behavioral and emotional problems in childhood (Degangi et al., 1993; DeGangi et al., 2000; Briggs-Gowan et al., 2006). Moreover, children with dysregulation disorders might have low self-efficacy and difficulties in developing a clear sense of identity (DeGangi, 2017) and are more at risk of developing both externalizing and internalizing disorders (Maestro et al., 2012) – particularly mood disorders, conduct disorders, substance abuse disorders and suicidal behaviors (Holtmann et al., 2011), as well as personality disorders (Halperin et al., 2011).

As for eating disorders (EDs), the few available longitudinal studies (Bryant-Waugh et al., 2010; Ammaniti et al., 2012; Lucarelli et al., 2018; Herle et al., 2020) have found a prevalence of homotypic continuity patterns from childhood to adolescence. During childhood, EDs tend to remain stable (Farrow and Blissett, 2012). Studies (Ammaniti et al., 2012) found that early anorexia is a risk factor for a subsequent diagnosis of anorexia nervosa as well as for anxiety, depression, and behavioral disturbances from infancy to childhood [see also (Lucarelli et al., 2018)]. Another longitudinal study (Kotler et al., 2001) found that bulimia during early adolescence was associated with a nine-fold increased risk for subsequent bulimia in late adolescence and a twenty-fold increased risk for bulimia nervosa in adulthood. Late adolescent bulimia nervosa was associated with a thirty-five-fold increase in risk for adult bulimia nervosa. Calam and Waller (1998) found that bulimic symptoms in early teenage years are a risk factor for subsequent bulimic behaviors, while restrictive symptoms predict later purging behaviors. Drive for thinness and body dissatisfaction, two core characteristics of EDs, show high homotypic continuity across development (Waszczuk et al., 2019). Instead, bulimia symptoms tend to be less stable and less correlated with the other two symptoms (*ibidem*). Herle et al. (2020) have recently shown that childhood overeating is a significant risk factor for adolescent binge eating disorder, while persistent undereating predicts anorexia nervosa in adolescent girls. Persistent picky eating is associated with greater anorexia nervosa risk in both girls and boys. Although the risk of developing an ED is created by a complex interplay between genetic, biological, psychological, and environmental factors, research has shown the presence of shared etiological pathways for EDs (Hilbert et al., 2014; Bakalar et al., 2015), as well as how EDs symptoms in early childhood and/or adolescence represent a significant risk for having an eating disorder later in life.

## 7. Homotypic and heterotypic continuity of personality traits and disorders

Both internalizing and externalizing disorders may represent significant precursors of personality disorders (Bernstein et al., 1996; Lewinsohn et al., 1997, 1999; Krueger, 2002).

Classic theories of personality have tried to resolve the dilemma of stability versus change in personality development. Some authors argued that predicting adult personality from childhood behaviors is not possible (Lewis, 1998). However, there is evidence that personality traits do originate early in life, with continuity patterns identified across childhood, adolescence, and adulthood (Caspi, 1998; Roberts and Del Vecchio, 2000), and that personality traits are often observed from an early developmental stage (Levy et al., 1999; Belsky et al., 2012; Fortunato et al., 2021, 2022a).

A longitudinal study that assessed the personality traits of 975 children from age three found that behavioral qualities observed during early childhood are linked to personality functioning at eighteen years old (Caspi and Silva, 1995) and twenty-six years old (Caspi et al., 2003). “Under-controlled” (i.e., irritable, impulsive) children maintain emotional liability traits in adulthood; “inhibited” children maintain over-controlled and non-assertive personality styles; “confident” and “well-adjusted” children become adults characterized by assertiveness and adaptive behaviors. Other studies found a correlation between impulsiveness in pre-school children, behavioral problems in school-age and antisocial behaviors in adolescence (Moffitt, 2003; Bergman et al., 2009; Calkins and Keane, 2009; Lahey and Waldman, 2017). In addition, behavioral disorders such as ADHD, ODD, and other conduct problems are related to antisocial behaviors; their presence represents a significant risk factor for developing disordered personality traits (Caye et al., 2016; Moffitt, 2018; Wertz et al., 2018). Withdrawal behaviors and shyness in early childhood are related to the presence of avoidant personality traits in adolescence (Eggum et al., 2009), and impulsive and externalizing behaviors during development predict the presence of a borderline personality disorder later in life (Stepp et al., 2012).

Other studies (Komsi et al., 2006) point out that temperament shows significant continuity from infancy to middle childhood. Although homotypic continuity in personality traits is most likely to be evident only after puberty, it is possible to assess the consistent developmental trajectories of some temperamental characteristics. Childhood mental disorders can be as stable as adult ones (Cohen et al., 1993; Putnam et al., 2008), and it is well known that the presence of a mental disorder during childhood increases the risk of showing a mental disorder in adolescence or young adulthood (Copeland et al., 2013). Although more research in this area is needed, the findings cited above support the hypothesis that personality disorders may originate from emerging maladaptive personality traits already evident in preschoolers. Moreover, research about non-pathological personality traits has found that the Big Five dimensions remain stable from childhood to adulthood (Shiner and Caspi, 2003). Such personality traits might become pathological due to transactional processes and the influence of environmental factors (van den Akker et al., 2016). Having a personality disorder in adolescence increases the risk of having the same personality disorder or other disorders in adulthood (Caspi, 1998; Kasen et al., 1999). Positive environmental experiences may reverse this trend (Kasen et al., 2009; Kasen and Cohen, 2009). A longitudinal study has shown that the presence of an MDD during childhood increases the odds of developing a dependent, antisocial, passive-aggressive, and histrionic personality disorder by more than 13, 10, 7, and 3 times, respectively (Kasen et al., 2001). This effect was independent of age, sex, socioeconomic status, the presence of adverse childhood experience, preexisting personality disorder in teenage years, and other childhood or adolescent mental disorders. The same study has also shown that the presence of a disruptive disorder during childhood increases the odds of developing a schizoid, narcissistic, and antisocial personality disorder (Kasen et al., 2001).

More longitudinal studies are needed to further investigate emerging personality patterns in childhood and how their evolution in adolescence and adulthood could lead to the development of personality disorders. However, some elements of continuity have already been found from childhood to adulthood (Fortunato and Speranza, 2018). Obsessive, depressive, and paranoid personality traits show homotypic continuity across the lifespan. Avoidant, dependent, borderline, histrionic, narcissistic, antisocial, schizotypal, and schizoid personality traits show heterotypic continuity patterns. These traits may partly overlap, and they can be difficult to distinguish in childhood; therefore, it is vital to shed light on personality functioning during childhood and develop assessment tools to capture its complex dynamics.

## 8. Toward an understanding of continuity: the role of attachment

The importance of the caregiver-child relationship as a protective or risk factor for development is well-known in the literature (Cicchetti, 1984; Sroufe and Rutter, 1984; Emde and Sameroff, 1989; Carlson and Sroufe, 1995; Emde, 2018; Dollberg and Keren, 2020; Farina et al., 2020; Speranza et al., 2020; Quintigliano et al., 2021; Yirmiya et al., 2022; Quintigliano et al., 2023). It is thus essential to consider this relationship when investigating psychopathology continuity. Attachment deserves particular attention among the developmental processes believed to underlie the continuity of psychopathological patterns. Attachment patterns tend to remain stable from infancy through adulthood (Waters et al., 2000). They can promote adaptive strategies or lay the groundwork for maladaptive behaviors, which may lead to the development of mental health disorders (Ma, 2006). Therefore, researchers have investigated the role of insecure or disorganized attachment in atypical development and psychopathology. Longitudinal studies (Sroufe et al., 1999, 2010; Sroufe, 2005) have demonstrated that insecure or disorganized attachment patterns (particularly the latter) are a significant predictor of maladaptive behaviors.

Attachment research also highlighted correlations between insecure or disorganized attachment in infancy and global indices of psychopathology (Cassidy and Mohr, 2001). Specifically, attachment disorganization increases the likelihood of psychopathology in adolescence (Carlson, 1998; Brown and Wright, 2003), and it is related to both internalization and externalization disorders, as well as to other conditions such as personality disorders (Lorenzini and Fonagy, 2013) and dissociative phenomena (Ogawa et al., 1997).

It seems that the results concerning attachment and psychopathology cannot be easily compared, primarily due to differences in both conceptualization and assessment of attachment dimensions. Moreover, longitudinal studies in this area are scarce. In the following sections, we will review some research highlighting the role of attachment in psychopathology since we believe that considering attachment patterns as affective regulation strategies, it is possible to identify to what extent a minimizing (avoidant attachment) or amplifying (resistant attachment) strategy might contribute to symptomatic expression and its continuity across development (Dozier et al., 2008).

### 8.1. Attachment and internalizing disorders

Children with insecure attachment exhibit more internalizing symptoms at 5 years old than secure ones (Pierrehumbert et al., 2000). Other studies showed that insecure attachment significantly predicts depression during adolescence (Duggal et al., 2001) and that anxious and avoidant attachment patterns predict depression and anxiety in teenage years (Lee and Hankin, 2009). However, a longitudinal study found that anxious-resistant attachment in infancy significantly predicted later anxiety disorders, while avoidant attachment did not (Warren et al., 1997). Yet other studies (Groh et al., 2012; Madigan et al., 2013) found the opposite trend: avoidant attachment correlated significantly, however moderately, with internalizing problems, while resistant attachment did not.

In their review, Brumariu and Kerns (2010) suggest that the mixed results regarding attachment and anxiety symptoms during development could be explained by the fact that few studies have considered the attachment disorganization dimension. In a later study, these authors have shown that attachment disorganization, but not avoidant or resistant attachment, was correlated with childhood anxiety (Kerns and Brumariu, 2014). Bureau et al. (2009) have shown that attachment disorganization predicts subsequent depressive symptoms in childhood, while attachment insecurity does not.

### 8.2. Attachment and externalizing disorders

Avoidant attachment is related to aggression and delinquency in boys (Renken et al., 1989). In addition, a longitudinal study (Aguilar et al., 2000) found that adolescents with early-onset and persistent antisocial behaviors were more likely to have been classified as insecure-avoidant. Similar results were found by Keller et al. (2005).

There is even more robust evidence regarding disorganized attachment as a predictor of later externalizing behaviors. Several studies have linked disorganized attachment in infancy with childhood externalizing behaviors (Lyons-Ruth, 1996; Shaw et al., 1996; Lyons-Ruth et al., 1997; Munson et al., 2001; Vondra et al., 2001; Futh et al., 2008; Bohlin et al., 2012; Forslund et al., 2020). This link appears more substantial than the one between externalizing symptoms and avoidant or resistant attachment (Shaw et al., 1996; Lyons-Ruth et al., 1997; Fearon et al., 2010). Disorganized attachment in infancy predicts aggression in school-aged children (van IJzendoorn et al., 1999) and is a significant risk factor for the development of ODD (Forslund et al., 2020). Such findings could be explained by the fact that behaviors associated with disorganized attachment give rise to a ‘controlling’ style, characterized either by role-reversed behaviors or punitive behaviors toward others (Main and Hesse, 1990). Children classified as controlling are more likely to show conduct problems (Solomon et al., 1995) or both externalizing and internalizing problems (Moss et al., 2004, 2006).

### 8.3. Attachment and other mental disorders

According to the PDM-2 (Lingiardi and McWilliams, 2017), considering attachment can foster our understanding of the child’s emerging personality (Bizzi et al., 2022; Fortunato et al., 2022b). An ambivalent attachment pattern is related to dependent and histrionic personality traits; the presence of a disorganized attachment in childhood, combined with the presence of other risk factors, could lead to the development of borderline personality disorder (Fortunato and Speranza, 2018). Caregivers who fail to meet their children’s attachment needs expose them to a substantial risk for psychopathology, especially for personality disorders. The role of attachment disorders in childhood as a risk factor for the development of a personality disorder is well-recognized; most data concern borderline personality disorder (Fonagy et al., 1996; Levy et al., 2011; Bogdan et al., 2014). A study (Rosenstein and Horowitz, 1996) found that adolescents with a dismissing attachment were more at risk of having conduct disorders, substance abuse disorders, and narcissistic or antisocial personality disorders or traits. Adolescents with preoccupied attachment are more likely to have an affective disorder, obsessive–compulsive, histrionic, borderline, or schizotypal personality disorder, and self-reported avoidant, anxious, and dysthymic personality traits. These data are in line with the aforementioned evidence linking separation anxiety in infancy and personality disorders (Silove et al., 2011; Goodman et al., 2013), as well as other mental disorders (Goodwin, 2003). There is also evidence of a link between attachment and dissociative experiences, typical of borderline personality disorder (Lorenzini and Fonagy, 2013). A longitudinal study (Carlson, 1998) revealed that children with a history of disorganized attachment were more likely to develop dissociative symptoms in adolescence. Further analyses on the same sample showed that disorganized attachment in infancy is strongly associated with dissociative symptoms (Ogawa et al., 1997).

## 9. Conclusions and suggestions for upcoming studies

The reported patterns of continuity in psychopathology suggest the need for early detection of its first clinical signs in infancy to implement strategies targeted at intervention and prevention (Cicchetti, 1984). Unfortunately, most studies have investigated only scholar age, not focusing on infancy or pre-schoolers (Kasen and Cohen, 2009). Studies are also often limited to specific populations (e.g., white, middle class; Ruchkin and Schwab-Stone, 2003). Finally, although the presence of homotypic and heterotypic continuity patterns across the lifespan has been largely demonstrated, further investigation is needed to understand the mechanisms that produce and maintain these trajectories.

Mechanisms underlying heterotypic patterns are indeed still under debate. The reviewed literature suggests that a given disorder may increase the risk of developing another if they share a common vulnerability factor. On the other hand, heterotypic patterns might occur by chance––in this case, however, continuity between them would not have possessed such strong empirical foundations (Ferdinand et al., 2007). Associations between disorders may also be mediated by comorbidity. Indeed, some heterotypic patterns disappear when comorbidities are considered. For this reason, some authors argue that the actual prevalence of heterotypic continuity is inflated (Angold et al., 1999). Other authors (Costello et al., 2003; Cummings et al., 2014) have found that heterotypic continuity persists even when controlling for comorbidity. A longitudinal study (Lahey et al., 2014) has shown that heterotypic patterns persist even after correcting homotypic ones. Various studies have focused on the influence of genetic and neurobiological factors: although the role of genetics has been acknowledged by most models on continuity (Rutter et al., 2006), it seems to play a role as a risk or mediating factor (Sroufe, 2009). As noted by Caspi et al. (2003), since many psychological characteristics such as temperamental qualities or personality traits are strongly related to genetic influences, these have previously been considered as able to explain continuity patterns. Such influences explain primarily how individual differences originate; however, continuity also appears to be strongly influenced by environmental factors.

Although it has not been thoroughly investigated in this paper, it is essential to mention the role of sex and gender in continuity. Some studies have shown that the prevalence of psychopathological disorders in childhood does not differ between sexes (Costello et al., 2003). However, some differences between the two sexes are present during pre-adolescence (Costello et al., 2003; Copeland et al., 2013), when depression becomes more common among females. Moreover, girls are more likely to present eating and affective disorders, while boys are more likely to have conduct and substance disorders (Reinke and Ostrander, 2008). Also, depression is often comorbid with CD in girls and substance use in boys (Lahey et al., 2000). However, more studies are needed to clarify the role of sex and gender in explaining dimorphic manifestations and their underlying processes.

Furthermore, the construct of continuity has relevant implications for diagnosis. The data reviewed in this paper support the utility of adopting a dimensional approach to mental disorders classification, especially during development. We must consider a complex interweave of factors to fully understand each form of psychopathology and its evolution over time. Adopting a dimensional approach could help clinicians and researchers to bridge the gap between clinical complexity and the need for empirical and methodological validity (Lingiardi and McWilliams, 2015). Biological and genetic dispositions, emotional regulation patterns, and the quality of early relationships with caregivers are all elements that influence the individual’s psychological functioning ––reflected through and organized in personality–– and are therefore all factors that should take into consideration both in psychological assessment and in studies investigating developmental trajectories of psychopathology (Lingiardi and McWilliams, 2017). An important gap in this area is the one regarding personality traits and disorders from childhood to adulthood. Considering personality functioning more thoroughly will allow clinicians and researchers to understand how seemingly different behaviors, symptoms, and disorders can evolve during development. It is essential, for example, to consider how individuals respond to their mental sufferance, how they give meaning to what happens to them, and if they believe to possess some degree of *agency* over it – all factors that influence how pathology will develop over time. Early adverse experiences profoundly impact self-representations and the quality of intimate relationships over time (Speranza et al., 2021), thus influencing personality development (Fortunato and Speranza, 2018; Riccardi et al., 2020). At the same time, a constitutional vulnerability to psychopathology might be amplified by a lack of adequate support by caregivers in childhood. In the famous nature versus nurture debate, it is essential to remember how personality represents a fundamental mediating factor of psychopathology. Since personality functioning can heighten or reduce the effects of environmental experiences, it can shape the characteristics, course, and stability over time of mental suffering.

In conclusion, knowing the homotypic and heterotypic continuity trajectories of mental disorders and being able to conceptualize them within a dynamic and comprehensive assessment frame can help clinicians and researchers better understand how psychopathology manifests itself during development. Recognizing the developmental trajectories of different mental disorders allows us to recognize them from the first subclinical signs, as well as to hypothesize how they will develop over time. This process is essential both for prevention and intervention – and can significantly help us formulate clinical hypotheses on the etiopathogenetic mechanisms underlying various disorders.

## Author contributions

AS: conceptualization and writing–review and editing. ML and IS: writing–original draft. AF: writing–review and editing. All authors contributed to the article and approved the submitted version.

## Conflict of interest

The authors declare that the research was conducted in the absence of any commercial or financial relationships that could be construed as a potential conflict of interest.

## Publisher’s note

All claims expressed in this article are solely those of the authors and do not necessarily represent those of their affiliated organizations, or those of the publisher, the editors and the reviewers. Any product that may be evaluated in this article, or claim that may be made by its manufacturer, is not guaranteed or endorsed by the publisher.
